# Visualizing spike activity during neuronal network development

**DOI:** 10.1186/1471-2202-15-S1-P209

**Published:** 2014-07-21

**Authors:** Nicholas Vachon, Thomas R Kiehl, Charles Bergeron

**Affiliations:** 1 Analytics Lab, Albany College of Pharmacy and Health Sciences, Albany, NY, 12208, USA; 2 Neural Stem Cell Institute, Rensselaer, NY, 12144, USA

## 

**Figure 1:**
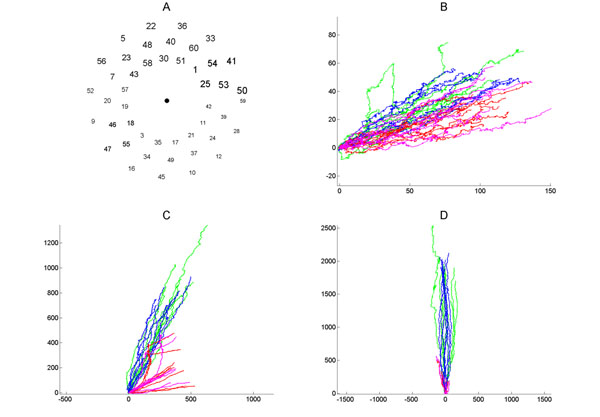
Development of an in vitro culture. **A.** Mapping of electrodes to angles. Larger numbers indicate electrodes with higher spike detection. **B.** Trails describing spontaneous (red and pink) and stimulated (green and blue) spike activity at 4 div; both axes represent arbitrary distance units. **C.** 13 div. **D.** 24 div.

We are interested in explaining neuronal network development through visualizations that summarize trends in large data. We utilized previously-recorded spiking patterns of embryonic rat cortex cells grown on multielectrode arrays [1]. We present results for batch 1 culture 3. Recordings were divided into 100 17.7 s intervals (the time required to sequentially stimulate each electrode at 0.3 s intervals). In our representation, each trail depicts an interval. The first 50 intervals recorded spontaneous activity (1-25 in red, 26-50 in pink); the last 50 intervals, activity in response to electrical stimulation (51-75 in green, 76-100 in blue). Each trail begins at the origin, and moves by 1 unit in a direction determined by the electrode detecting it. Longer trails indicate more active intervals. After 4 days in vitro (div) (Figure 1B), activity is scattered and minimal. At 10 div, spiking is more frequent (Figure 1C). Spontaneous activity shows some consistency, as does the stimulated activity, but they differ. At 24 div, spontaneous and stimulated patterns are similar (trails are oriented in the same direction); stimulation provokes many more spikes (Figure 1D). We continue to assess the value of these visualizations in terms of biological characterization.
